# Reconstruction Using Frozen Autograft for Disseminated Phosphaturic Mesenchymal Tumor of the Humerus after a Pathological Fracture

**DOI:** 10.1155/2019/3015675

**Published:** 2019-09-04

**Authors:** Hiroshi Kobayashi, Toshihide Hirai, Naohiro Makise, Tetsuo Ushiku, Nobuaki Ito, Minae Koga, Masachika Ikegami, Yusuke Shinoda, Toru Akiyama, Sakae Tanaka

**Affiliations:** ^1^Department of Orthopaedic Surgery, The University of Tokyo Hospital, 7-3-1 Hongo, Bunkyo-ku, Tokyo 113-8655, Japan; ^2^Department of Pathology, The University of Tokyo Hospital, 7-3-1 Hongo, Bunkyo-ku, Tokyo 113-8655, Japan; ^3^Department of Nephrology and Endocrinology, The University of Tokyo Hospital, 7-3-1 Hongo, Bunkyo-ku, Tokyo 113-8655, Japan; ^4^Department of Orthopaedic Surgery, Saitama Medical Center, Jichi Medical University, 1-847 Amanuma, Omiya-ku, Saitama 330-8503, Japan

## Abstract

Phosphaturic mesenchymal tumors (PMTs) that cause tumor-induced osteomalacia (TIO) occur commonly in the bone with a small and nonaggressive appearance. Here, we report the case of a 67-year-old man with disseminated PMT in the humerus after a pathological fracture. Liquid nitrogen was used as an adjuvant therapy after curettage of the tumor, and the frozen autograft, using a pedicle freezing method, conserved the function of the shoulder joint. To our knowledge, this is the first case of a disseminated PMT in the bone that was treated with a frozen autograft, and this treatment method may be effective for cases in which curettage for PMT in the bone would be inevitably inadequate.

## 1. Introduction

Tumor-induced osteomalacia (TIO) is a rare paraneoplastic syndrome caused by phosphaturic mesenchymal tumors (PMTs) that secrete fibroblast growth factor 23 (FGF23) and inhibit the reabsorption of phosphorus in the proximal renal tubule [1]. PMTs can originate anywhere in the body, and one-third of tumors occur in the bone [2]. PMTs in the bone represent various imaging features and tend to infiltrate intertrabecular regions and the adjacent cortical bone [3]. To treat TIO, the tumor should be completely excised as minute residual tumor fragments may continue to produce sufficient amounts of FGF23 to cause osteomalacia on the basis of putative derangement in the serum phosphate sensing mechanism in PMTs. Therefore, the preferred surgical treatment methods for PMTs in the bone are extended curettage and segmental resection [3].

Frozen autografts are often used for reconstruction following the wide resection of malignant bone tumors in Japan [4]. Liquid nitrogen at −196°C destroys tumor cells by freezing, thereby devitalizing tumor cells by inducing ice crystal formation and cell dehydration [4]. After treatment with liquid nitrogen, frozen bones are used as autografts to reconstruct bone defects, with both osteoconductive and osteoinductive effects [4]. Most treatments with autografts are performed using two-sided osteotomies, with a high risk of nonunion. However, the frozen autograft technique can be used in “pedicle freezing,” which requires only one-sided osteotomy and maintains continuity with the remaining bone without freezing to avoid the risk of nonunion and enabling immediate return of viable cells [5].

To our knowledge, there is only one report of locally aggressive PMT in the bone with pathological fracture [6]; however, there are no reports of disseminated PMT in the bone. In this report, we describe a case of disseminated PMT of the humerus after pathological fracture. A frozen autograft using liquid nitrogen as an adjuvant therapy was effective for PMT in the bone. Reconstruction using a frozen autograft after resection of the tumor of the humerus conserves the function of the shoulder joint. Furthermore, bone union after the autograft is achieved using the pedicle freezing technique, even if the bone quality is poor owing to osteomalacia.

## 2. Case Presentation

**Figure 1:**
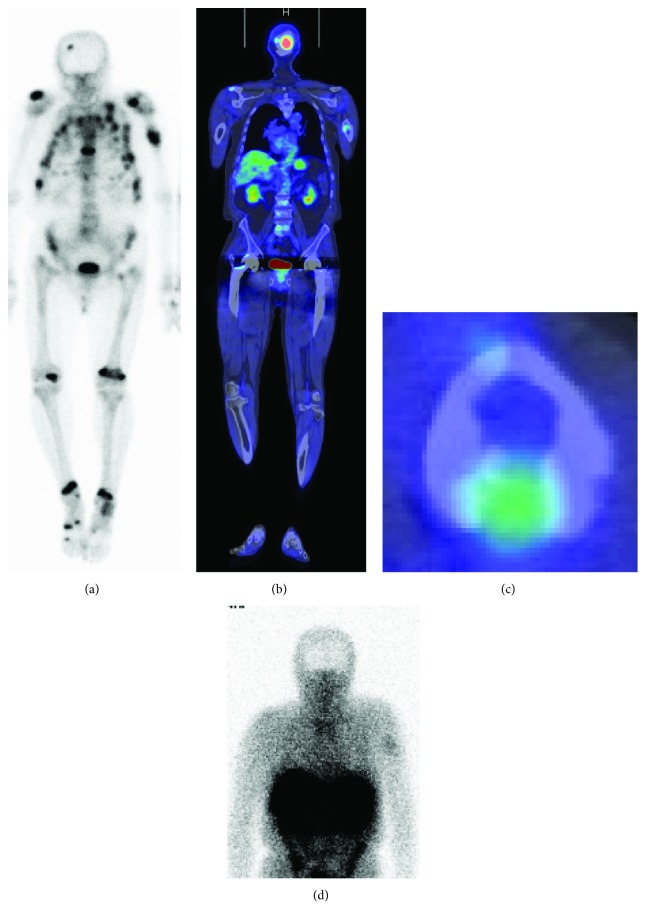
(a) Bone scintigraphy demonstrating multiple areas of abnormal uptake, including the left humerus, distal bilateral femur, distal bilateral tibia, thoracic vertebra, and multiple ribs. (b, c) Positron emission tomography/computed tomography (PET/CT) showing increased uptake at the left humerus. (d) OctreoScan revealing increased uptake at the left humerus as with PET/CT.

**Figure 2:**
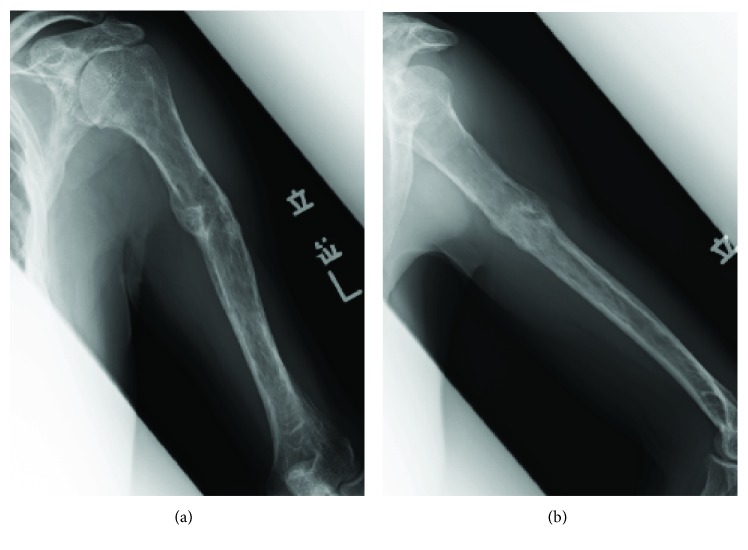
Preoperative radiograph of the left humerus ((a) anteroposterior view; (b) lateral view) showing varus deformity at the healed fracture site and osteolytic lesion with endosteal scalloping at the diaphysis.

**Figure 3:**
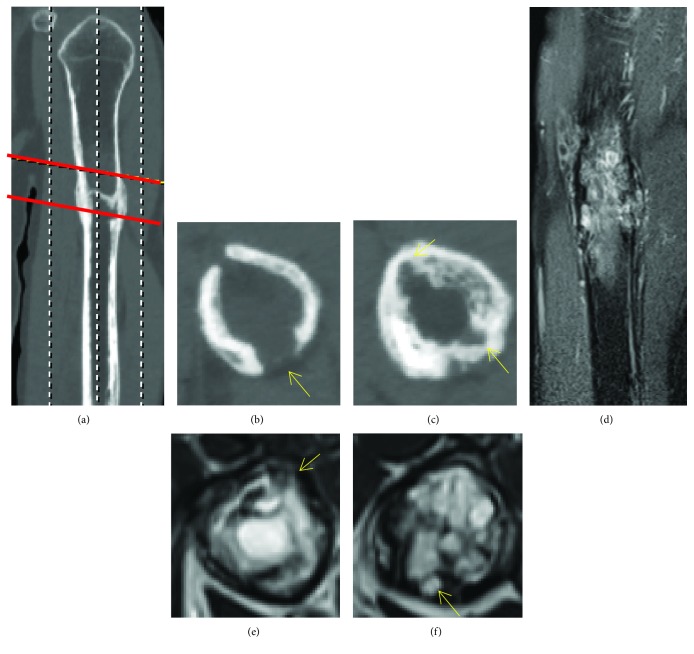
(a) Computed tomography of the left humerus showing ((b) upper line level of sagittal view) partial cortical destruction (yellow arrow) and ((c) lower line level of sagittal view) erosion (yellow arrow). (d) T2-weighted fat suppression magnetic resonance image (MRI) of the left humerus showing longitudinal invasion of the tumor, a nearly 7 cm extent in the diaphysis. Axial view of T2-weighted fat suppression of MRI revealing tumor invasion to the (e) extracortical lesion (yellow arrow) and (f) intracortical lesion (yellow arrow).

A 67-year-old man was referred to our hospital with suspicion of TIO. Eight years before presentation, he noticed bilateral coxalgia, and two years later, he underwent total hip arthroplasty (THA) because of a diagnosis of femoral neck fracture at a hospital near his home. Two years after THA, he experienced left arm pain and was diagnosed with a pathological fracture of the humerus. Incisional biopsy was diagnostic of a “benign tumor,” and the humeral fracture was treated conservatively. One year prior to presentation, he went to several hospitals because of the multiple fractures and decreased body height. Osteomalacia was suspected because of low serum phosphate levels. In our hospital, the patient was referred to the endocrinology department, and TIO was suspected because of an elevated FGF23 level (249.3 pg/ml; reference range: 10–50 pg/ml). Venous sampling of FGF23, positron emission tomography/computed tomography (18F-FDG PET/CT), and somatostatin receptor-based functional scans (OctreoScan®) identified the suspected causative lesion of TIO in the left humerus (Figure 1). Plain radiographs showed a varus deformity of the humerus and a slightly osteolytic lesion with endosteal scalloping in the diaphysis of the humerus (Figure 2). Left humerus computed tomography showed partial cortical erosion and destruction (Figure 3(a)–3(c)). Left humerus magnetic resonance imaging (MRI) scans revealed a nearly 7 cm sized lesion in the diaphysis with intracortical and extracortical lesions but without soft tissue dissemination (Figure 3(d)–3(f)). These findings indicated that the tumor was disseminated to the intracortical and extracortical lesions after a pathological fracture.

**Figure 4:**
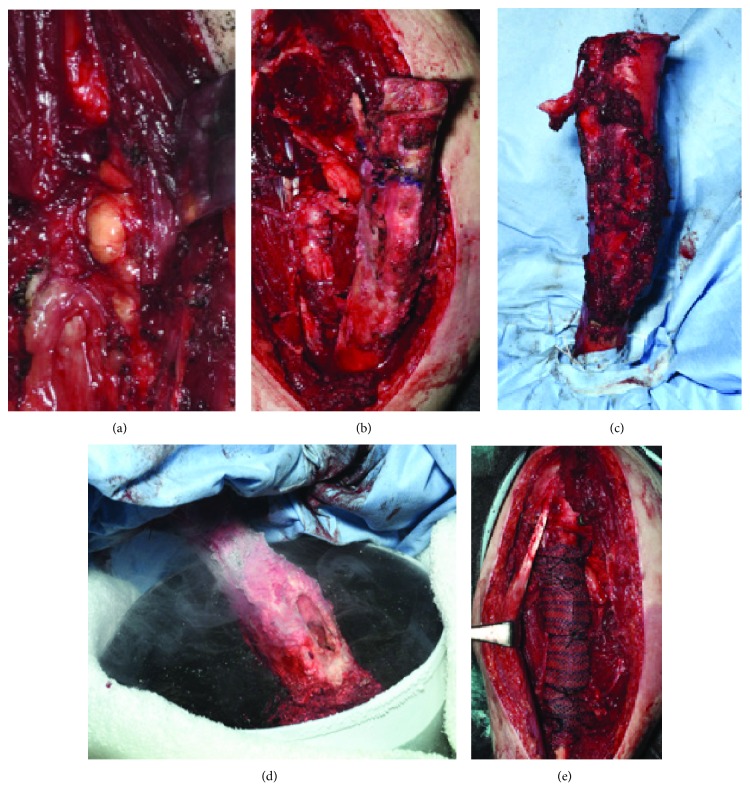
Intraoperative findings. (a) Extracortical lesion observed. (b) One-sided osteotomy 1 cm apart from the proximal end of the tumor. (c) Isolation from other surgical areas with drapes. (d) Pedicle freeing. (e) Wrapping the humerus after internal fixation and resuturing the deltoid muscle, pectoralis major, biceps, and triceps.

**Figure 5:**
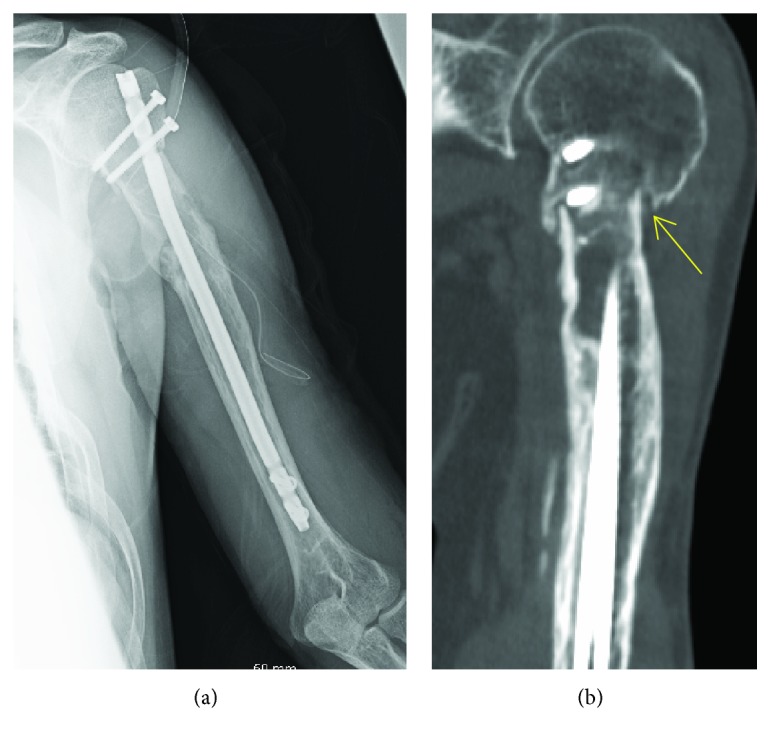
(a) Postoperative radiograph of the left humerus representing internal fixation of the osteotomy site after correction of varus deformity using an intramedullary nail. (b) Postoperative CT at 8 months revealing bone union at the osteotomy site.

A surgical procedure was performed through a longitudinal incision from the deltopectoral groove to the lateral side of the upper arm. Muscles attached to the humerus, including the deltoid, were detached from the humerus, and the well-demarcated extracortical tumor was identified at the anterior aspect of the humerus continuing to the medullary cavity (Figure 4(a)). Instead of two-sided osteotomy of the humerus, we osteotomized the proximal portion of the humerus with a 1 cm margin from the tumor in the medullary cavity, preserved the radial nerve, and detached muscles without extended soft tissue resection from the humerus with a 6 cm safety margin from the distal end of the tumor to prevent normal soft tissue freezing (Figure 4(b)). The humerus, including the tumor and the 1 cm margin, was isolated from other surgical areas with drapes (Figure 4(c)). Curettage was performed, taking care not to push the tumor into the distal medullary cavity. The proximal humerus with a 1 cm margin from the distal end of the tumor was frozen in liquid nitrogen for 20 minutes while replenishing the liquid nitrogen repeatedly (Figure 4(d)) followed by defrosting at room temperature for 15 minutes and in distilled water for 10 minutes. The proximal portion of the humerus was osteotomized obliquely to correct the alignment of the humerus and fixed with an intramedullary nail with autologous bone grafting from the ilium, filling the bone defect and enhancing bone healing (Figure 5(a)). Detached muscles, including the deltoid muscle, triceps medialis, and pectoralis major, were sutured to the polypropylene mesh wrapping the humerus (Figure 4(e)).

**Figure 6:**
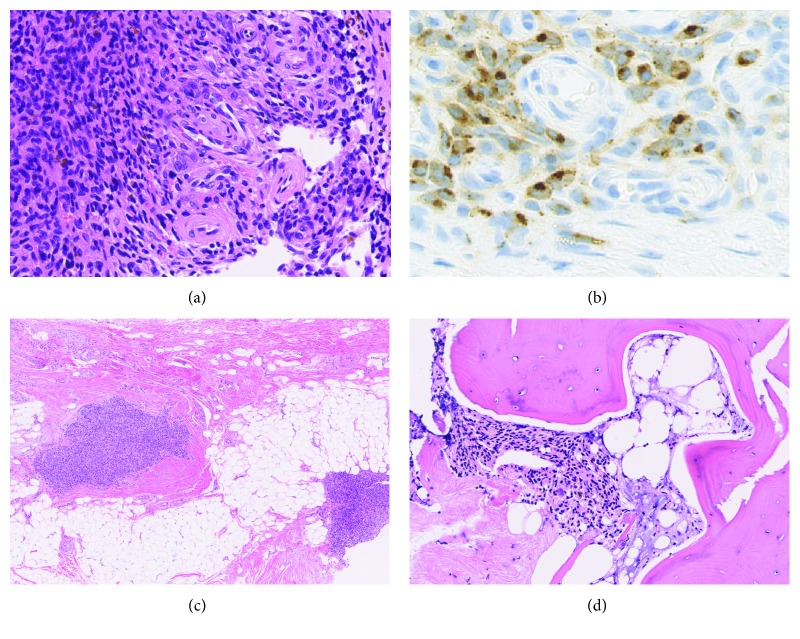
Histopathology of the tumor. (a) The tumor was composed of sheet-like proliferation of uniform oval-shaped tumor cells in a fibrous background with prominent capillary-sized vessels. (b) The tumor was focally positive for FGF23 with a punctate perinuclear staining pattern, while blood vessels were negative. (c) Soft tissue invasion of the tumor cells. (d) Intracortical invasion of the tumor cells.

On histological examination, the tumor was composed of sheet-like or vague storiform proliferation of uniform oval-shaped tumor cells in a fibrous background with prominent capillary-sized vessels (Figure 6(a)). Hemorrhage, hemosiderin deposition, and scattered multinucleated giant cells were observed. Immunohistochemically, the tumor was focally positive for FGF23 (Figure 6(b)). The diagnosis of PMT, mixed connective tissue variant, was made. Soft tissue invasion (Figure 6(c)) and intracortical invasion (Figure 6(d)) were identified in the extraosseous and intraosseous lesions, respectively.

Phosphorous levels returned to normal 5 days after the surgery. After the surgery, the shoulder was immobilized with an arm sling for 3 weeks, followed by physiotherapy. Symptomatic improvement was noted over the next several months. The patient was able to walk with crutches. Back-out of the locking screw inserted in the humeral head was observed two months after the surgery; however, bone union was observed at 8 months (Figure 5(b)). Neither local recurrence nor low phosphate levels were observed 1 year later, with active shoulder flexion and abduction at 110° and 90°, respectively. The ISOLS/MSTS score was 28/30. There was some functional disability of the left shoulder and arm; however, the patient was able to use his left arm for activities of daily living.

## 3. Discussion

We presented the case of a patient with disseminated PMT of the humerus after pathological fracture and its successful treatment using the frozen autograft technique.

PMTs in the bone represent various imaging features, and most radiologic images published in the literature show PMTs as small and nonaggressive in appearance [3]. A few cases of PMTs in the bone with aggressive features have been reported, and a case of PMT in the humerus with invasion into the medullary cavity and pathological fracture has also been reported [6]. Our case is notable for the reason that it is an extremely rare case in which the tumor disseminated into intracortical and extracortical lesions after a pathologic fracture. Complete tumor excision is important as residual tumor fragments may produce enough FGF23 to osteomalacia due to a disturbance in the serum phosphate sensing mechanism. Therefore, extended curettage or segmental resection are recommended to excise the tumor completely. A similar case of locally aggressive PMT in the humerus was treated with proximal humeral resection and reconstruction with a reverse total shoulder arthroplasty [6]. Reverse total shoulder arthroplasty is a good option for reconstructing shoulder function after resecting the proximal humerus, including the humeral head; however, the functional outcome of composite reverse shoulder arthroplasty after resecting malignant tumors is inferior to that of cases of rotator cuff injury because the detachment of muscles, including the deltoid muscle, is temporarily required [7]. Furthermore, as previously reported, early loosening of endoprosthesis is warranted in patients with TIO due to poor bone quality [8]. In our patient, the tumor did not invade the humeral head; therefore, we opted to perform autograft reconstruction by recycling the bone while preserving the humeral head to prevent the need for endoprosthesis.

In our patient, liquid nitrogen was an efficient adjuvant therapy after curettage for PMT in the bone. We usually use ethanol as an adjuvant therapy after curettage for PMT in the bone, as is the case with intermediate bone tumor, including giant cell tumors. However, we selected liquid nitrogen treatment, according to malignant bone tumors, because inadequate curettage was guaranteed in the context of this patient's disseminated intracortical lesion. There are two other methods of adjuvant therapy in recycling bone for malignant bone tumors: irradiation [9] and pasteurization [10]. By comparison to these other methods, liquid nitrogen has the merit of being uncomplicated without any specific equipment, maintaining osteoinductive properties, and being able to use the “pedicle freezing” method [4]. The conventional technique for liquid nitrogen treatment is “free freezing,” following the process of extracorporeal soaking in liquid nitrogen after en bloc resection of the bone, and returning to its original site for reconstruction. This technique is associated with a relatively high risk of nonunion, because it involves a two-sided osteotomy. Conversely, “pedicle freezing” requires osteotomy of one side only; that is, the treated bone remains in continuity with the normal bone. We applied this method to reduce the nonunion rate, even if the osteomalacia of the treated bone was relatively worse than that of normal bone. Kimura et al. [11] reported that, in four cases, bone union after pedicle freezing required 8, 9, 9, and 14 months involving the resection of the proximal humerus while preserving the humeral head. In our case, the duration was equivalent to that report, namely, 8 months, suggesting that a frozen autograft is a feasible treatment for PMT in the bone with respect to cytotoxicity to tumor cells, osteoinductivity, and osteoconductivity.

In summary, this is the first report of a rare case of disseminated PMT of the humerus after pathological fracture. The frozen autograft technique, especially the pedicle freezing method, is an effective method for disseminated cases of PMT.
